# Community Vulnerability: Measuring the Health Situation of a Population After COVID-19 Through Electronic Health Record Indicators

**DOI:** 10.3390/healthcare13010068

**Published:** 2025-01-02

**Authors:** Andrea Sierra-Ortega, Alexandra González-Aguña, Marta Fernández-Batalla, Enrique Monsalvo-San Macario, Blanca Gonzalo de Diego, Lourdes Jiménez-Rodríguez, José María Santamaría-García

**Affiliations:** 1 Meco Health Centre, Community of Madrid Health Service (SERMAS), 28880 Madrid, Spain; blanca94gd@gmail.com (B.G.d.D.); chesantgar@hotmail.com (J.M.S.-G.); 2 Research Group MISKC, Department of Computer Science, University of Alcala, 28805 Madrid, Spain; alexandra.gonzalez@salud.madrid.org (A.G.-A.); marta.fdezbatalla@gmail.com (M.F.-B.); enrique.monsalvo@hotmail.com (E.M.-S.M.); lou.jimenez@uah.es (L.J.-R.); 3 Santa Cristina University Hospital, Community of Madrid Health Service (SERMAS), 28009 Madrid, Spain; 4 Northern Assistance Directorate, Primary Care Assistance Management, Community of Madrid Health Service (SERMAS), 28035 Madrid, Spain; 5 Juan de Austria Health Centre, Community of Madrid Health Service (SERMAS), 28804 Madrid, Spain

**Keywords:** community health status indicators, diagnosis of health situation, health, health vulnerability, nursing social vulnerability index, primary care nursing, primary healthcare

## Abstract

**Background:** The COVID-19 pandemic made people face the fact that we are all vulnerable. This vulnerability can be measured through the Basic Variables of Care (BVC) using the Care Vulnerability Index (CVI). Health systems work with indicators that nurses can consult to understand the care and health situation of their population. These indicators provide valuable information on the vulnerability of the population. **Objective:** to determine the level of community vulnerability of a population group using health indicators from the computerized clinical records of Primary Care. **Methods:** observational, retrospective study from March 2023 to January 2024, with a sample of 2106 people assigned to a nurse at the Meco Health Centre (Madrid, Spain). Phases: selection of dashboard indicators, linkage to BVC, score assignment, population adjustment and calculation of the CVI. **Results:** The sample of indicators selects 18 out of 376 included in the Specific Dashboard; each indicator is related to 6 to 10 BCVs, with different rank values. Each score is adjusted by a Vulnerability Correction Factor according to the number of people included in the indicator. Finally, the population-adjusted CVI scores 1.95 points (percentile 37.90). **Conclusions:** community vulnerability is an essential tool in community health diagnostics and can be measured through health indicators that reflect the care situation of a population group at a given time, including changes in the situation in the face of health crises such as the COVID-19 pandemic.

## 1. Introduction

Nearly five years after the first cases of people with COVID-19, this health problem continues to be of interest for clinical care and research. The 2020 pandemic and its subsequent consequences, in all spheres of life, have highlighted the fragility of health systems and the importance of establishing sociopolitical and health strategies to protect the most vulnerable. In this sense, the concept of vulnerability and the tools that make it possible to measure this vulnerability in individuals and population groups is key to inform clinical prioritization decisions.

In a society marked by knowledge management, these tools must collect and analyse the data available in health system dashboards to return meaningful information that distinguishes individuals, families and/or communities according to their level of vulnerability.

In this sense, the general objective of this research is to determine the level of community vulnerability of a population group by means of health indicators from the electronic health record of Primary Care.

### 1.1. Impact of the COVID-19 Pandemic on Population and Healthcare

People with COVID-19 disease caused by the severe acute respiratory syndrome coronavirus 2 (SARS-CoV-2) show mainly respiratory symptoms, but it also affects the neurological or digestive system. An impact on people’s health that has varied is the speed of contagion, as well as in the expression of the disease—its severity and mortality [1,2].

The COVID-19 pandemic emergency was declared in March 2020 and the end of the emergency was decreed in May 2023; however, there were already cases in December 2019, the evolution has followed epidemiological wave patterns and even today there are periods of increased infection among the population [3,4,5].

Epidemic waves are periods of time when there is a sustained increase in cases during a sustained period (upswing) followed by a sustained decrease during another period (downswing). Maintenance over time and a substantial number of cases are necessary to identify a wave, as opposed to a one-time spike, changes in inclusion criteria and/or errors in the reporting system [5,6].

Worldwide, information is available on a total of four waves per COVID-19; within Europe, at least three, and in Spain, six waves have been recognized up to the start of this research study. The data used to determine the trends and limits of the rising and falling periods of each wave are based on the records of the Carlos III Institute. Specifically, the Community of Madrid reached an incidence in the last wave of 159.5 cases/10,000 inhabitants and a positivity rate of 23.1% [7,8,9,10,11].

No new wave notifications are currently available, but the end of the COVID-19 disease, which continues to be present today, cannot be estimated [12,13,14].

COVID-19 is a disease that continues to be considered a population health risk, with periods of increased risk of infection requiring community healthcare and seasonal vaccination campaigns similar to influenza vaccination campaigns [15,16,17,18,19].

These healthcare and vaccination campaigns prioritize vulnerable population groups that are defined according to age criteria and concomitance with certain chronic health processes or care situations [20,21].

### 1.2. Care Vulnerability Index (CVI)

The framework of the Knowledge Model about Person Care, the Vulnerability Model defines vulnerability as a quality inherent to people, from birth to death, and as the possibility of being hurt or receiving physical or moral injury [22,23].

This vulnerability is dynamic and linked to the need and competence for self-care. Changes in the level of vulnerability result from an increase in need, a decrease in competence or a combination of both [23].

The Care Vulnerability Index (CVI) is a tool for calculating the level of vulnerability of individuals based on the Vulnerability Model developed by Fernández Batalla [23].

The definition of each BVC is shown in Table 1.

Each of the BVCs can take three rank values that are scored with 0, 0.5 and 1 point, from the lowest to highest level of vulnerability, respectively. These scores are combined into clusters and the scores are summed. The final result is a score between 0 (minimum vulnerability) and 15 points (maximum vulnerability) [23].

The CVI is defined by the BVCs that define the person and their vulnerability, in relation to the characteristics of the environment that represent the risk. The union of both, vulnerability and risk, gives rise to the predisposition to suffer care problems.

The CVI has been used to calculate individual or family group vulnerability, but it was never adapted to analyse community vulnerability.

In this sense, the study of community vulnerability (sometimes called social vulnerability) is usually linked to variables related to the living conditions of specific groups, the characteristics of the environment, the climate, or even access to and use of the health system. All of them are characteristics of the context in which one lives. However, in this research, community vulnerability will be linked to inherent conditions as people [23,24,25].

### 1.3. Primary Care Health Indicators

Twenty years ago, the Community of Madrid designed a plan to improve Primary Care (the first level of healthcare system in Spain) that included the availability of a homogeneous information system with the capacity to integrate different levels of responsibility and components of care. This plan was based on indicators, as predefined variables that reflect a complex reality, which were designed by integrating them with data extraction from different sources such as electronic clinical records [26]. This information system took the form of a Balanced Scorecard in a digital environment known as e-SOAP (electronic Primary Care Objectives Monitoring) [27,28].

The e-SOAP scorecard transfers to the organization and its professionals the strategic management where actions are developed and implemented to achieve the established objectives. These objectives are common to all Primary Care nurses and include strategic lines of the health system, program contract with annual objectives and other areas of interest [27,28].

A Balanced Scorecard highlights objectives, makes monitoring visible and allows performance evaluation. These indicators allow comparison of the same level of responsibility over time, as well as between different units at the same level of analysis [27,28].

Since its implementation, e-SOAP has evolved and increased the number of indicators. Currently, e-SOAP has a Specific Dashboard oriented to clinical management to improve care processes and added value in intermediate health outcomes. Each indicator offers four levels of aggregation: Community of Madrid, Healthcare Management (division of the territory for healthcare), Health Centre Director and Healthcare Professional. In this sense, the professional can access the information of their assigned patients (population quota) and export the aggregated and anonymous data to a file in Excel^®^ format [28].

Subsequently, the professional can analyse the information reported by the indicators to learn about the healthcare situation of his or her population. This information can be used under models to know the vulnerability of the population or the impact of their care intervention on intermediate health outcomes [2,23,27,28,29].

## 2. Materials and Methods

This study is observational, retrospective, cross-sectional and uses Deductive Care Methodology (DCM) to draw inferences of logical relationship between indicators and BVCs, as well as to work from an algebraic mode with the scores in the calculation of the CVI [23,30].

### 2.1. Scope

This study was carried out in the Community of Madrid (Spain), at Primary Care, in the East Care Directorate, within the Healthcare Centre Meco. This multidisciplinary team covers the municipality of Meco and, in addition, Camarma de Esteruelas, Los Santos de La Humosa and Valdeavero. The total population is 27,790 people [31,32].

### 2.2. Period

The data collected correspond to the period March 2023–January 2024.

### 2.3. Study Sample

The sample of the study population are the persons assigned to a Family and Community Nurse Specialist at Healthcare Centre Meco. This professional has a total of 2106 people assigned to him as of 25 February 2024, corresponding to the municipality of Meco, but also to the other municipalities mentioned above.

**Figure 1:**
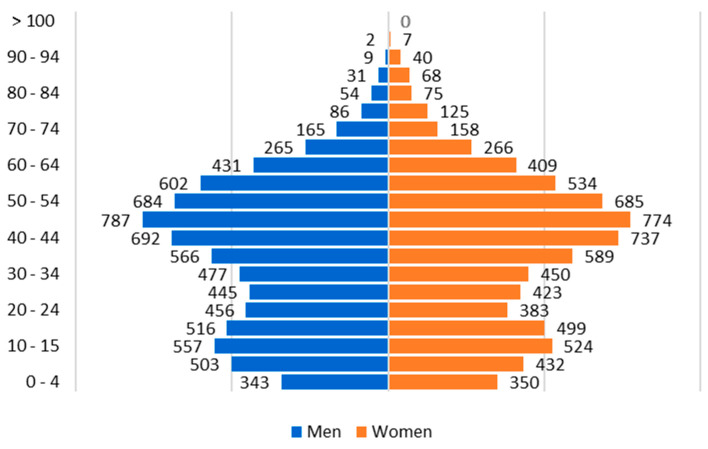
Meco population pyramid.

Meco is a village in the East of the Community of Madrid that in 2022 had 14,856 people (49.33% men and 50.67% women). The population is somewhat younger compared to the Madrid region because it has an aging degree (population >64 years in relation to total population) of 9.37% and youth degree (population 0–14 years in relation to total population) of 17.18% [33]. The population pyramid is shown in Figure 1.

The foreign population represents 18.32%, similar to other municipalities in the area and the region of Madrid. Unemployment amounts to 4.82%, similar to Madrid. The working people are mainly dedicated to the distribution and hotel sector, although in Meco, there are mainly residential housing areas, offices and, in third place, areas dedicated to industry [33].

The study sample includes people from all over the municipality without limitations of any condition because the Primary Care nursing service is a reference for people of any age or condition.

The sample size calculation on the total population of Meco (14,856 people) is 638 people for a confidence interval (IC) of 99% and a margin of error of 5%. Thus, the study sample includes 2106 people, which ensures representativeness. In addition, the age and sex distribution are similar to the population pyramid of Meco. The study sample under 15 years of age is 13.58%, between 15 and 64 years of age is 76.73% and from 65 years of age is 9.69%. The distribution by sex is similar, with 49.91% men and 50.09% women.

### 2.4. Process

This study carries out a methodological process that applies theory and practice. From the abstraction level, this study uses a Care ontology (Knowledge Model about Person Care) as a framework through a model (Vulnerability Model). This model is put into clinical practice through an assessment instrument (CVI).

On the other hand, from the clinical level in a real situation, this study uses a population sample from a town in Madrid where the first level of care uses a single electronic clinical history per person with a structure common to the entire region. This information system is linked to a dashboard with indicators common to all health centres that allow monitoring relevant information on more than 3 million Madrid residents.

**Figure 2:**
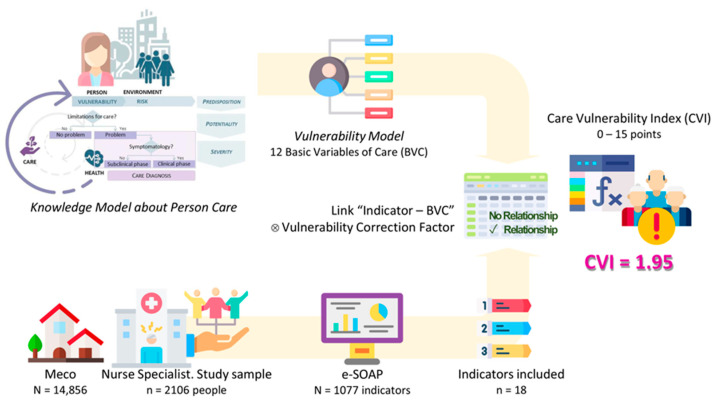
Methodological process of this study.

The summary of the methodological process of this study is shown in Figure 2.

#### 2.4.1. Selection of e-SOAP Indicators

The first step is to select the indicators through which the data reflecting the vulnerability situation of the study sample are obtained, its means, to identify indicators related to the BVC.

The indicators are selected from the e-SOAP Balanced Scorecard (*n* = 1077 indicators) and within the specific Dashboard (*n* = 376 indicators) they are filtered with the following selection criteria [34]:

Phase I Screening. The objective of this first phase is a screening to identify the indicators of the sample universe for the study. This phase is divided into two rounds. Round 1: Exclude indicators not directly linked to the clinical activity of the reference nurse. These support units are Palliative Home Care Support Team (PHCST), Midwife, Physiotherapy and Oral and Dental health. Round 2: Exclude indicators not operational at the time of study. Indicators that are in e-SOAP but do not have measurement data for the study period.

Phase II Eligibility. This phase aims to identify the objectives specific to the purpose of the study, so three rounds of exclusion are applied. Round 3: Exclude indicators on the efficiency of the professional’s activity that do not report on the health outcomes of the person but report on health activity processes. For example, implementation of care plans, follow-up control of patients with a specific chronic disease. Round 4: Exclude indicators linked to processes regulated by regulations that have an obligation of equal care for all persons and, therefore, do not allow discriminating the person’s level of care. For example, follow-up of polymedicated program, vaccination schedule. Round 5: Exclude indicators associated with the COVID-19 process that arise specifically ad hoc during the pandemic because they have shown changes in the definition of the indicator, as well as in the ability to identify the actual population data assigned to the referring nurse professional, because individuals were cared for in centralized resources or at their place of work.

Phase III Inclusion. The last phase aims to determine the study sample through the definitions of the indicators in order to minimize the computational cost of calculating the CVI and thus optimize the efficiency of the study. This phase has only one round where four exclusion criteria are applied: indicators composed of two lamps; for example, percentage of people with arterial hypertension with ventricular hypertrophy, or percentage of people with ischemic heart disease and/or stroke with controlled blood pressure levels; indicators associated with administrative processes; for example, indicators on care plans for specific populations, frequentation of the health system; indicators associated with pharmacological treatment; for example, patients with heart disease treated with statins and indicators associated with healthcare processes. For example, good control, effective management, overuse of test strips for measuring blood glucose, pressure ulcers in bedridden persons.

#### 2.4.2. Relationship of the Indicators to the Basic Variables of Care

The sample of selected indicators is associated to each of the BVCs by means of a content analysis. This content analysis includes the definition of each BVC and the definition of the indicator to establish whether or not an indicator provides information on that BVC.

The first step is to create a Karnaugh table that shows the relationship between each indicator and BVC. Once the relationship is established, the study details the range value of the BVC to which the indicator is linked.

For example, the indicator “patients with arterial hypertension with age-adjusted blood pressure control” is related to the BVC “Physical limitation”, and, specifically, this indicator reports the range value “Compensated” physical limitation. However, the indicator “persons who engage in risky alcohol consumption” is related to the BVC “Physical limitation” and range value “Not compensated”.

At the end of this detailed relationship at the BVC rank value level, each indicator is linked to a vulnerability score according to the CVI. Continuing with the previous examples, the indicator “patients with arterial hypertension with age-adjusted blood pressure control” has a score of 0.5 and the indicator “Persons with risky alcohol consumption” has a score of 1; both out of a maximum of 1. This score reflects that persons with risky alcohol consumption are more vulnerable than persons with controlled hypertension, but both are higher than healthy people without chronic disease or toxic habits.

For this research, the BVC Age score (used as Life stage) requires adaptation because the indicators do not share the same life stage divisions according to years. In this sense, the study uses two rank values: Life Stage Childhood–Adolescence and Life Stage Youth–Elderly. The score for each stage is the arithmetic mean of the scores for the life stages it encompasses. The Childhood–Adolescence life stage scores 0.75 points and the Youth–Elderly life stage scores 0.25 points out of a maximum of 1 point as the highest level of vulnerability.

This step allows us to start working from the DCM, specifically, the Algebraic Mode.

The BVCs, their rank values and the score for the CVI calculation are shown in Table 2.

#### 2.4.3. Weighting by Vulnerability Correction Factor

The selected indicators refer to a part of the total population sample studied, i.e., the indicators report on healthcare outcomes of a group of people in the sample, but do not report on the total number of people assigned to the nurse (*n* = 2106 people).

In addition, these specific groups are different for each indicator, so the scores from the previous phase must be adjusted. Furthermore, each indicator is measured on more than one occasion in the period under study, so it is necessary to estimate a population mean for each indicator.

The number of people included in each indicator (the proportion of the total population) is the average of the number of people included in the indicator at each cut-off during the nine-month study period. For example, if an indicator has three measurements in the study period (March 2023–January 2024), the study adds the number of people included in each measurement and divides by three.

This correction factor is called the Vulnerability Correction Factor (VCF) and allows estimating a weight adjusted to the actual population analysed.

The population-adjusted weight (adjusted score) is obtained by multiplying the score given in the previous phase by the proportion of the population included in the indicator in relation to the total study sample.

Population-adjusted weight = (CVI points of the indicator × VCF)/total number of people in the study sample.

#### 2.4.4. Calculation of Vulnerability Index

The last phase of the study calculates the CVI.

First, the average score of each BVC is calculated using the arithmetic mean of the scores (once the VCF has been applied) of all the indicators that are associated with the BVC.

The score for each DVI cluster is then calculated [23]:

Cluster 1 = ((Life Stage + Developmental Status)/2) × 5

Cluster 2 = ((Perception of gender limitation + Sociocultural integration)/2) × 1

Cluster 3 = ((Family care system + Individual care system)/2) × 3

Cluster 4 = ((Physical limitations + Cognitive limitations + Sensory limitations)/3) × 4

Cluster 5 = ((Environmental Factors + Material Resources + Time Resource)/3) × 2

The final CVI score is the sum of the scores of all clusters, with the final range of scores being between 0 points (Lowest vulnerability) and 15 points (Highest vulnerability)

CVI = Cluster 1 + Cluster 2 + Cluster 3 + Cluster 4 + Cluster 5 [23].

### 2.5. Data Analysis

The research applies the Excel^®^ program for the electronic notebook for data collection, score analysis and calculation of the CVI.

The process meets validity and reliability criteria. The methods used include the collection of data from the entire sample, from an official data source, with anonymous data and applying correction factors to the score according to the number of persons in the sample included in each indicator. In this sense, the data are obtained from a single source of data, official and unique for the entire Community of Madrid, with direct access to the data and the need for processing for the study.

### 2.6. Ethical Considerations

Researchers followed the guidelines of good ethical and scientific practice established in The European Code of Conduct for Research Integrity [35].

The ethical principles and those enunciated in the Helsinki Statement on Health in All Policies and in the Oviedo Convention for the protection of human rights and fundamental freedoms were considered. Although the data collected were anonymous, in whatever could occur, reference was made to data protection legislation Organic Law 3/2018 on Personal Data Protection and guarantee of digital rights, and Regulation (EU) 2016/679 of the European Parliament and of the Council of 27 April 2016 on the protection of natural persons with regard to the processing of personal data [36,37,38,39].

The research did not collect or identify individual persons (researchers did not make queries from the Autonomous Community Personal Identification Code) at any time during the consultation carried out in the clinical information system referring to the electronic clinical record. Researchers could not identify personal data because they had group-level general data as a group of persons attended by a health professional.

The Research Ethics Committee of the Príncipe de Asturias University Hospital assessed and approved the research study on 31 January 2024 (Code: OE 06/2024).

## 3. Results

The results of this study are shown below in order:

### 3.1. Indicators Selection

After the analysis and screening process, the sample has a total of 18 indicators.

**Figure 3:**
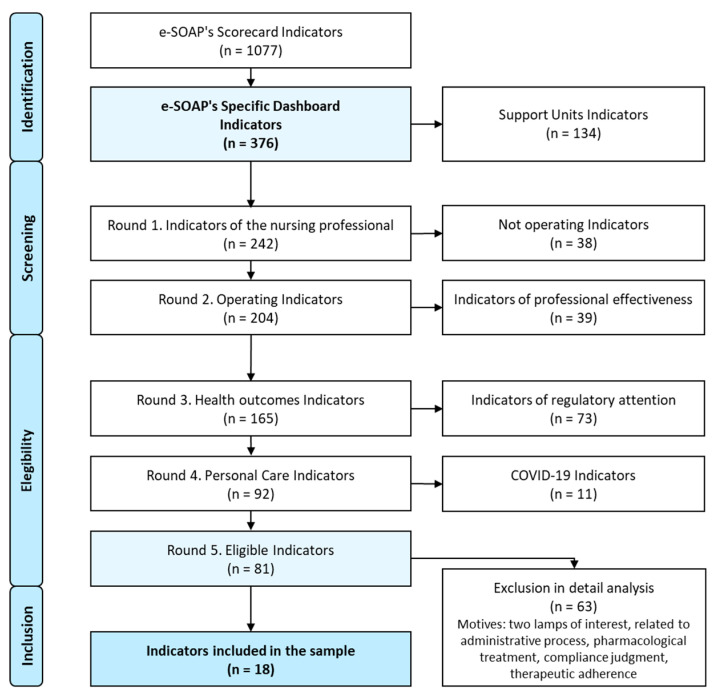
Flowchart of the selection of indicators for the study sample.

The selection process is shown in Figure 3.

The indicators included in the study sample are detailed in Appendix A and are summarized as follows:

1. People with severe or total functional impairment (Barthel ≤60) with care plans.
2. Management of hypercholesterolemia in secondary prevention of ischemic heart disease
3. Chronic patients in health institutions with assigned NI (≥70 years)
4. Patients with ischemic heart disease and controlled blood pressure levels
5. Diabetic patients with adequate age-adjusted glycated haemoglobin control.
6. Patients with diabetes and controlled blood pressure levels
7. Patients with chronic kidney disease and controlled blood pressure levels adjusted for age and albuminuria.
8. Patients with stroke and controlled blood pressure levels.
9. Patients with heart failure and controlled blood pressure levels.
10. Patients with arterial hypertension with age-adjusted blood pressure control
11. People with healthy eating
12. Persons with risky alcohol consumption.
13. Smokers
14. People who practice physical exercise
15. Obese children
16. Overweight children
17. Children with healthy eating
18. Children who practice physical exercise

### 3.2. Relationship of the Indicators to the Basic Variables of Care

The relationship between each indicator and the BVC uses the indicator label, the formula, the definition and, especially, the inclusion and exclusion criteria that appear in the indicator sheets.

One criterion widely used by indicators is age. Indicators usually make explicit to which type of population they are limited by age. Therefore, the sample is not the entire nursing population, but only the adult, paediatric or elderly population. This condition already establishes a relationship between the indicator and the BVC “Life Stage” and the results reflect which life stage, and which score it corresponds to for the calculation of the CVI. On the other hand, the indicators do not provide information on the BVC “Developmental State” and therefore the results show this BVC without relationships to indicators.

Regarding the BVC “Sociocultural integration”, the people included in these indicators were considered to be enculturated, because they had attended the health system and therefore communication and understanding between the person and the health professional was possible.

Regarding the BVC “Family system of care”, it relates to any person who is dependent for his/her care and needs the help of another person to meet his/her needs. Further, on the BVC “Individual system of care”, all the indicators give information on some sphere of care for these people, whether it is about living habits or about the degree of control of their basic disease.

Another area is healthcare limitations. BVC “Physical limitations”: all indicators that refer to diseases that affect the physical or physiological level of the person, as well as those related to mobility limitations. BVC “Cognitive limitations”: all indicators that refer to diseases or life situations that affect the ability to understand and make decisions. BVC “Sensory limitations”: all indicators referring to diseases or health processes that alter any of the senses (sight, hearing, taste, smell, touch).

As for the BVC “Environmental factors”, it is favourable because the geographical area of study does not have limitations for self-care. The area has electricity, water, healthy streets, green areas, security systems (local police), supermarkets, schools, libraries and parks; they are not municipalities that are undergoing natural disasters or are at war, in which case it would be considered a hindering environment for self-care.

Finally, the BVC “Material resources” and BVC “Time resource” are considered favourable based on the municipality’s data. Meco is within Madrid and has a public health system with a health centre in the area and a nearby hospital, as well as other resources for social care or dependent people or people with different types of needs. The population is generally adult, active and there are no unemployment or similar problems that could be interpreted as a difficulty of resources or time.

The Karnaugh chart reflecting the relationship between each indicator and the BVC rank value is shown in Table 3.

Among the results, it is noteworthy that the BVC *Sex* shows no relationship (NR) with any indicator. This BVC is included in the initial analysis but is not part of the CVI because it does not condition the possibility of suffering harm. Perception of limitation associated with gender is the BVC that is included in the CVI.

Once the BVC Sex is removed from the table, the relationship is transformed from natural language to the corresponding score according to the CVI, thus moving to an algebraic methodology that allows the calculation to grade the levels of vulnerability. The result of the indicator–BVC relationship with the scores for the calculation of the CVI is shown in Table 4.

### 3.3. Weighting by Vulnerability Correction Factor (VCF)

The scores obtained in the previous phase are adjusted by means of the VCF that considers the difference in the number of persons represented by each selected indicator.

This correction factor and the adjusted scores are shown in Table 5.

### 3.4. Calculation of Care Vulnerability Index (CVI)

Finally, this study calculates the CVI by applying the formula presented in the methodology that groups the BVCs into five clusters, which have a different score from 1 to 5 points and a final result between 0 and 15 points.

Table 6 shows the CVI calculated with the maximum scores and population-adjusted weighted scores.

The final result is a maximum CVI of 5.15 points out of 15, taking the maximum values as a reference (see Table 3), and a CVI adjusted to population weight of 1.95 points out of 15, taking the values adjusted by VCF as a reference. In this sense, if 5.15 points is the maximum vulnerability of the sample studied, a CVI of 1.95 corresponds to the 37.90 percentile, that is, 5.15 is the maximum in the vulnerability percentile of this population (100th percentile) and the study reveals a population with a CVI in the 37.90th percentile.

## 4. Discussion

This study achieves its main objective to determine the level of community vulnerability of a population through health indicators.

For this purpose, the research applies the CVI, which is a tool that, unlike other similar publications, integrates a nursing model centred on the person, called the Vulnerability Model [22,30].

Fernández Batalla’s Vulnerability Model is based on previous care models that have demonstrated their clinical validity: Orem’s Self-care Deficit Theory and Santamaría García’s Care Gravity Index. In addition, this Vulnerability Model has been applied and validated with national and international studies, with different populations and healthcare circumstances. These studies are available as impact publications [22,23,30].

In this regard, similar publications on community vulnerability coincide in the type of variables under study.

Redd et al. proposed in 2003 the Community Health Index Status Model as an assessment method for the United States and Canada that includes data from two domains: at the individual level and at the community level. The model data are along the same lines as the Vulnerability Model. At the individual level, data are collected on age, sex, gender, income, employment status, chronic conditions such as diagnosis of personal health problems or family history, health behaviours such as food, smoking, drinking alcohol, exercise, sleep habits, belt use, dental care or screening programs. At the community level, data are collected on community life, personal safety, access to the healthcare system, satisfaction with the service, illegal drug use, homelessness, poverty and other global health determinants such as demographics, economics or community health [40].

Bowie and Lawson (2018) employ a Vulnerability Index to assess the health needs of a homeless community. The index uses a questionnaire of 34 short questions divided into five sections: demographic information, housing, health system, health conditions and social history. Included within this tool is a subscale to identify high mortality risk, which includes data such as emergency use in the last three months, hospitalization or emergency use in the last year, age over 60 years, HIV/AIDS, kidney disease, liver disease, cold weather injury and trimorbidity status [41].

Finally, Fallah-Aliabadi et al. (2022) conducted a systematic review to establish a set of social vulnerability indicators in the context of the COVID-19 pandemic. The indicators are classified into seven categories: Household, community composition, Race, minority status and language, Socioeconomic status, Community health status, Public health infrastructures, Education, Information, technology and communication [24].

Further, and from an analysis of the Knowledge Model about Person Care [22], some of the characteristics of the previous studies could be considered as part of the risk of the environment for a person, or, by contrast, as part of the vulnerability if analysed as a community.

Moreover, one of the difficulties in estimating the vulnerability of a community is the source of data. Health systems use different scorecards (and specific dashboards), have different objectives and, therefore, different indicators. In addition, the periodicity or frequency of measurement of these indicators varies. Vulnerability studies for groups face the difficulty of not having the same model of care, health lines and objectives, as well as differences in the availability of scorecards and their indicators. This difficulty does not appear in the present study because the research is carried out in the Primary Care setting of Madrid, which has a common regulatory framework, with action plans, scorecards and homogeneous indicators for all the Health Centres that serve the entire population of Madrid [27,28].

### 4.1. Limitations of the Study and Future Lines

This research has some limitations that should be pointed out.

Firstly, the data source is a Specific Dashboard common to the entire Primary Care level in Madrid; however, the sample of a single professional in a limited period of time is used [27,28,34].

This condition implies a clinical bias because each nurse prioritizes and manages the care of the population according to their professional criteria, being able to perform more or less follow-up of certain health processes [42].

The limitation of a period of time can also lead to a bias in the data if it coincides with certain circumstances; for example, collection during the pandemic period would result in low levels of registrations for attention to chronic processes and high levels for acute respiratory processes. The study was conducted over a nine-month period and was expected to cover a large part of the reference population sample.

However, in the Community of Madrid Health Service, all individuals are assigned a nurse and a physician at the Primary Care level. This professional is the reference for selecting the sample in a specific health centre. However, the clinical history is not unique to a professional. The clinical history is unique and the same for each person throughout life and for all the professionals who provide care in Primary Care. In this sense, although the study uses a study sample with reference to a specialist nurse, the indicators studied are the result of all the health interventions of the person throughout life, with any health professional and in any of the health centres in Madrid. The recording biases that appear if only what is written by one professional is studied are minimized because the study collects records from different professionals and at different times of life.

On the other hand, the indicators may have a bias because they are based on records of the population attended at the health centre. This means that, although the population sample is 2106 people, not all people attend the health centre. One profile of people who do not routinely go to the health centre are healthy working adults, because they do not perceive a need or reason (life processes or health problems) to seek healthcare. This subgroup of healthy adults may be underrepresented, which would lead to results of greater vulnerability because the indicators arise from data on people who have healthcare needs that they cannot meet on their own and demand healthcare. This circumstance can be considered globally for all health centres, and not as an exclusive characteristic of this study. However, as it represents to a lesser extent the less vulnerable population (healthy adult person), it is foreseeable that the CVI offers a result somewhat higher than the real vulnerability. From a clinical point of view, in terms of safety and quality of care, this circumstance (where the CVI offers a somewhat higher vulnerability result) allows planning human resources and material resources from a perspective aimed at identifying the worst situation of vulnerability and burden of care [23,43,44,45].

In the future, these limitations offer new lines of research with studies in different population groups, or in the same group, but with annual cutoffs over time. Likewise, future studies should delve into the validation of the selected health indicators and evaluate whether the defined CVI would improve the number of indicators from a cost-effective point of view in order to design and implement a technological application of the community CVI.

### 4.2. Relevance to Clinical Practice

The CVI has a history of practical usefulness, but it has never been applied from a group or community point of view.

This study reflects the value of the CVI and the opportunity to work with standardized health indicators defined and available in all centres of a healthcare network that serves more than 6.5 million people of different nationalities.

The use of health indicators as a source of data for calculating community vulnerability shows that technological applications can be used effectively and efficiently even in adverse environments, for example, following health crises such as COVID-19.

Replication of the study with different population groups, in the same period of time, allows comparison of the level of vulnerability managed by each nurse or other health professionals. In addition, it provides a continuous programmed assessment of the level of vulnerability of population groups and communities, which can be used to predict the needs of the population in the face of new pandemics and to prioritize efforts in the distribution of health resources.

Likewise, replicating the study constantly over time makes it possible to evaluate and monitor the evolution of the population’s health, the effectiveness of the professional’s actions and the history of care in a community.

This should be the reference point for further integration of these health technologies in the design and delivery of post-pandemic and inter-pandemic healthcare, as it allows the identification of vulnerable populations and the prioritization of healthcare delivery to those most in need.

This is of great interest in the Primary Care and community healthcare setting, since care specialists such as family and community nurses can prioritize their clinical interventions towards care actions focused on health education, assistance or accompaniment of their population, depending on the level of vulnerability of the population to which they provide care.

The results of the CVI make it possible to identify which populations are more vulnerable and require more resources; or which professionals are assigned to the most vulnerable populations. From a management point of view, the calculation of the CVI enables the generation of dashboard systems, to homogenize the burden of care of the different professionals within the same health team or allows the most vulnerable populations to be assigned to the most highly trained specialist professionals.

The results of this study lay the basis for a significant transformation of healthcare following the COVID-19 pandemic, so that healthcare systems and their professionals can be better prepared to meet the future needs of the population after suffering global health crises such as COVID-19.

For all these reasons, the application of the CVI to a population makes it possible to determine its vulnerability situation, in order to specifically prioritize community health promotion, prevention and rehabilitation activities, at the health, economic and/or social levels in post-pandemic periods.

## 5. Conclusions

COVID-19 is still active today and, therefore, knowing the health situation of populations in post-pandemic times allows us to discover the new population needs, as well as the pending needs to be covered by the health system.

After a pandemic crisis situation such as COVID-19, the availability of updated data on the community health situation is key for health management in post-pandemic situations and subsequent strategic planning actions at different levels.

Vulnerability is an individual but also a collective concept where a group expresses a level of vulnerability inherent to its own individual and community characteristics.

The CVI, defined in previous studies at the individual or family level, can be calculated for an entire community. The knowledge model is the same in both cases because it uses the same Vulnerability Model and the same data source. A Specific Dashboard proves to be sufficient and adequate for obtaining the indicators provided by the data. Therefore, this community vulnerability can be monitored through the Specific Dashboard (included in e-SOAP) with a set of indicators available to all Primary Care nurses in Madrid (Spain).

The calculation of community vulnerability after the COVID-19 pandemic provides objective data on the prioritization of resources to the most vulnerable populations, at the health, economic and/or social levels.

The application of the CVI in post-pandemic and inter-pandemic situations also makes it possible to predict future population needs in the event of the emergence of new global health crises.

This is possible thanks to the availability of a sequence of health-vulnerability benchmarks with which to compare the health evolution of the population, and to be able to manage, plan and prioritize healthcare resources efficiently; since this study is carried out with standardized indicators, available and common to all professionals in a national health region, it is possible to replicate in those health services that have computerized medical records.

In conclusion, community vulnerability is the set of vulnerabilities of the people who make up a community. This definition assumes the relationship between two concepts: vulnerability and community.

Vulnerability is understood as the inherent quality of people to be injured or susceptible to physical or moral harm and it is particularly sensitive to health crises such as pandemics. This is because vulnerability in the field of health conditions the balance of people’s needs and competencies for their own care and the care of others, including the care of their environment.

But the study of vulnerability, as evidenced by COVID-19, must be carried out not only on an individual level, but also on a group or community level.

By community we mean a group of people, whether or not connected temporally or spatially, who share a common purpose, interest, values and principles, identified through common care habits, which have an impact on their health and life. The COVID-19 pandemic repositioned the value of the community health approach to address global health problems.

Therefore, the measurement and monitoring of community vulnerability, through the Community Vulnerability Index, is an essential tool in community health diagnoses and makes it possible to be prepared for future COVID-19 outbreaks or other global health crisis situations.

## Figures and Tables

**Table 1:** Basic Variable of Care definitions.

BVC	Definition
Life stage	Classification of people according to their maturational state from birth to old age.
Developmental State	Sequential physiological, psychological and moral changes during a person’s lifetime.
Perception of gender limitation	Perception of attitudes or behaviours that limit the capacity for self-care based on stereotyped social roles related to the internal experience of the gender role.
Sociocultural integration	Level of attachment that a person perceives with his or her current place of residence in the physical, emotional and functional context. This attachment should be reciprocal between the community and the person.
Family system of care	System of care that encompasses the knowledge, skills and attitudes that the family imparts to the person.
Individual system of care	A system of care that integrates knowledge, skills and attitudes to meet the needs and develops the capabilities of the individual.
Physical limitations	Difficulty walking from one place to another or mobility of any part of the body.
Cognitive limitations	Difficulty in mental processes related to learning, thinking, reasoning and judgment.
Sensory limitations	Difficulty in perceiving external and internal sensory stimuli to which the individual responds (sight, hearing, smell, taste and touch).
Environmental factors	Influence of the elements of the environment to condition in some way the person’s ability to exercise self-care.
Material resources	Amount of material resources that the person can have or seek for self-care.
Time Resource	Amount of time the person may have or seek for self-care.

BVC: Basic Variable of Care.

**Table 2:** Basic Care Variable (BVC), rank values and score for Care Vulnerability Index (CVI).

BVC	Rank Value	CVI (Points)
Life Stage	Childhood–Adolescence	0.75
	Youth–Elderly	0.25
Developmental State	Age appropriate	0
	Stagnant or undeveloped	0.5
	Regressive to age	1
Gender	Hindering	0.5
	Indifferent	1
Sociocultural Integration	Enculturated for self-care	0
	In the process of enculturation	0.5
	Acculturated or enculturated against self-care	1
Family System of Care	Comprehensive family support	0
	Partial family support (for health problems)	0.5
	No family support	1
Individual System of Care	Comprehensive healthcare	0
	Partial healthcare (in case of health problems)	0.5
	Indifferent to healthcare	1
Physical Limitations	No mobility limitation	0
	Compensated physical limitation	0.5
	Uncompensated physical limitation	1
Cognitive Limitations	No cognitive limitation	0
	Compensated cognitive limitation	0.5
	Uncompensated cognitive limitation	1
Sensory Limitations	No sensory limitation	0
	Compensated sensory limitation	0.5
	Uncompensated sensory limitation	1
Environmental Factors	Caregiver promoters	0
	Caring hinderers	0.5
	Incompatible with care	1
Material Resource	Adequate material resources	0
	Search for material resources is required	0.5
	Impossible availability of material resources	1
Time Resource	Adequate time resources	0
	Time search required	0.5
	Impossible availability of time	1

BVC: Basic Variable of Care.

**Table 3:** Relationship of the indicators with the rank value of each BVC.

Indicator	Life Stage	Sex	Developmental State	Gender	Sociocultural Integration	Family System of Care	Individual System of Care	Physical Limitations	Cognitive Limitations	Sensory Limitations	Environmental Factors	Material Resource	Time Resource
People with severe or total functional impairment (Barthel ≤ 60) with care plans	Youth–Elderly	NR	NR	NR	Enculturated	Partial	Indifferent	Uncompensated	Uncompensated	NR	Caregiver promoters	Adequate	Adequate
Management of hypercholesterolemia in secondary prevention of ischemic heart disease	Youth–Elderly	NR	NR	NR	Enculturated	NR	Partial	Compensated	NR	NR	Caregiver promoters	Adequate	NR
Chronic patients in health institutions with assigned NI (≥70 years)	Youth–Elderly	NR	NR	NR	Enculturated	Comprehensive	Partial	Compensated	NR	NR	Caregiver promoters	Adequate	Adequate
Patients with ischemic heart disease and controlled blood pressure levels	Youth–Elderly	NR	NR	NR	Enculturated	NR	Partial	Compensated	NR	NR	Caregiver promoters	Adequate	NR
Diabetic patients with adequate age-adjusted glycated haemoglobin control	Youth–Elderly	NR	NR	NR	Enculturated	NR	Partial	Compensated	NR	Compensated	Caregiver promoters	Adequate	NR
Patients with diabetes and controlled blood pressure levels	Youth–Elderly	NR	NR	NR	Enculturated	NR	Partial	Compensated	NR	Compensated	Caregiver promoters	Adequate	NR
Patients with chronic kidney disease and controlled blood pressure levels adjusted for age and albuminuria	Youth–Elderly	NR	NR	NR	Enculturated	NR	Partial	Compensated	NR	NR	Caregiver promoters	Adequate	NR
Patients with stroke and controlled blood pressure levels	Youth–Elderly	NR	NR	NR	Enculturated	Partial	Partial	Compensated	NR	NR	Caregiver promoters	Adequate	NR
Patients with heart failure and controlled blood pressure levels	Youth–Elderly	NR	NR	NR	Enculturated	NR	Partial	Compensated	NR	NR	Caregiver promoters	Adequate	NR
Patients with arterial hypertension with age-adjusted blood pressure monitoring	Youth–Elderly	NR	NR	NR	Enculturated	NR	Partial	Compensated	NR	NR	Caregiver promoters	Adequate	NR
People with healthy eating	Youth–Elderly	NR	NR	NR	Enculturated	NR	Comprehensive	NR	NR	NR	Caregiver promoters	Adequate	Adequate
Persons with risky alcohol consumption	Youth–Elderly	NR	NR	Hindering	Enculturated	NR	Indifferent	Uncompensated	Uncompensated	Uncompensated	Caregiver promoters	Search required	Adequate
Smokers	Youth–Elderly	NR	NR	Hindering	Enculturated	NR	Indifferent	Uncompensated	NR	Uncompensated	Caregiver promoters	Adequate	Adequate
People who practice physical exercise	Youth–Elderly	NR	NR	Indifferent	Enculturated	NR	Comprehensive	Compensated	Compensated	Compensated	Caregiver promoters	Adequate	Adequate
Obese children	Childhood–Adolescence	NR	NR	NR	Enculturated	Partial	Comprehensive	Compensated	NR	NR	Caregiver promoters	Adequate	Adequate
Overweight children	Childhood–Adolescence	NR	NR	NR	Enculturated	Partial	Comprehensive	NR	NR	NR	Caregiver promoters	Adequate	Adequate
Children with healthy eating	Childhood–Adolescence	NR	NR	NR	Enculturated	Comprehensive	Comprehensive	NR	NR	NR	Caregiver promoters	Adequate	Adequate
Children who practice physical exercise	Childhood–Adolescence	NR	NR	NR	Enculturated	Comprehensive	Comprehensive	Compensated	NR	NR	Caregiver promoters	Adequate	Adequate

BVC: Basic Variable of Care. NR: No Relationship.

**Table 4:** CVI score for each indicator—BVC ratio.

Indicator	Life Stage	Developmental State	Gender	Sociocultural Integration	Family System of Care	Individual System of Care	Physical Limitations	Cognitive Limitations	Sensory Limitations	Environmental Factors	Material Resource	Time Resource
People with severe or total functional impairment (Barthel ≤ 60) with care plans.	0.25	-	-	0.00	0.50	1.00	1.00	1.00	-	0.00	0.00	0.00
Management of hypercholesterolemia in secondary prevention of ischemic heart disease	0.25	-	-	0.00	-	0.50	0.50	-	-	0.00	0.00	-
Chronic patients in health institutions with assigned NI (≥70 years)	0.25	-	-	0.00	0.00	0.50	0.50	-	-	0.00	0.00	0.00
Patients with ischemic heart disease and controlled blood pressure levels	0.25	-	-	0.00	-	0.50	0.50	-	-	0.00	0.00	-
Diabetic patients with adequate age-adjusted glycated haemoglobin control.	0.25	-	-	0.00	-	0.50	0.50	-	0.50	0.00	0.00	-
Patients with diabetes and controlled blood pressure levels	0.25	-	-	0.00	-	0.50	0.50	-	0.50	0.00	0.00	-
Patients with chronic kidney disease and controlled blood pressure levels adjusted for age and albuminuria.	0.25	-	-	0.00	-	0.50	0.50	-	-	0.00	0.00	-
Patients with stroke and controlled blood pressure levels	0.25	-	-	0.00	0.50	0.50	0.50	-	-	0.00	0.00	-
Patients with heart failure and controlled blood pressure levels	0.25	-	-	0.00	-	0.50	0.50	-	-	0.00	0.00	-
Patients with arterial hypertension with age-adjusted blood pressure monitoring	0.25	-	-	0.00	-	0.50	0.50	-	-	0.00	0.00	-
People with healthy eating	0.25	-	-	0.00	-	0.00	-	-	-	0.00	0.00	0.00
Persons with risky alcohol consumption	0.25	-	1.00	0.00	-	1.00	1.00	1.00	1.00	0.00	0.50	0.00
Smokers	0.25	-	1.00	0.00	-	1.00	1.00	-	1.00	0.00	0.00	0.00
People who practice physical exercise	0.25	-	0.00	0.00	-	0.00	0.50	0.50	0.50	0.00	0.00	0.00
Obese children	0.75	-	-	0.00	0.50	0.00	0.50	-	-	0.00	0.00	0.00
Overweight children	0.75	-	-	0.00	0.50	0.00	-	-	-	0.00	0.00	0.00
Children with healthy eating	0.75	-	-	0.00	0.00	0.00	-	-	-	0.00	0.00	0.00
Children who practice physical exercise	0.75	-	-	0.00	0.00	0.00	0.50	-	-	0.00	0.00	0.00
Average	0.36	0.00	0.67	0.00	0.29	0.42	0.60	0.83	0.70	0.00	0.03	0.00
SD	0.21	0.00	0.58	0.00	0.27	0.35	0.21	0.29	0.27	0.00	0.12	0.00
Median	0.25	0.00	1.00	0.00	0.50	0.50	0.50	1.00	0.50	0.00	0.00	0.00

CVI: Care Vulnerability Index; BVC: Basic Variable of Care; SD: Standard Deviation.

**Table 5:** CVI score with population-adjusted weight after applying Vulnerability Correction Factor.

Indicator	VCF	Life Stage	Developmental State	Gender	Sociocultural Integration	Family System of Care	Individual System of Care	Physical Limitations	Cognitive Limitations	Sensory Limitations	Environmental Factors	Material Resource	Time Resource
People with severe or total functional impairment (Barthel ≤ 60) with care plans.	2.25	0.00	-	-	0.00	0.00	0.00	0.00	0.00	-	0.00	0.00	0.00
Management of hypercholesterolemia in secondary prevention of ischemic heart disease	19.25	0.00	-	-	0.00	-	0.00	0.00	-	-	0.00	0.00	-
Chronic patients in health institutions with assigned NI (≥70 years)	13.25	0.00	-	-	0.00	0.00	0.00	0.00	-	-	0.00	0.00	0.00
Patients with ischemic heart disease and controlled blood pressure levels	16.50	0.00	-	-	0.00	-	0.00	0.00	-	-	0.00	0.00	-
Diabetic patients with adequate age-adjusted glycated haemoglobin control.	91.50	0.01	-	-	0.00	-	0.02	0.02	-	0.02	0.00	0.00	-
Patients with diabetes and controlled blood pressure levels	80.50	0.01	-	-	0.00	-	0.02	0.02	-	0.02	0.00	0.00	-
Patients with chronic kidney disease and controlled blood pressure levels adjusted for age and albuminuria.	10.25	0.00	-	-	0.00	-	0.00	0.00	-	-	0.00	0.00	-
Patients with stroke and controlled blood pressure levels	24.00	0.00	-	-	0.00	0.01	0.01	0.01	-	-	0.00	0.00	-
Patients with heart failure and controlled blood pressure levels	4.50	0.00	-	-	0.00	-	0.00	0.00	-	-	0.00	0.00	-
Patients with arterial hypertension with age-adjusted blood pressure monitoring	237.25	0.03	-	-	0.00	-	0.06	0.06	-	-	0.00	0.00	-
People with healthy eating	1772.33	0.21	-	-	0.00	-	0.00	-	-	-	0.00	0.00	0.00
Persons with risky alcohol consumption	1772.33	0.21	-	0.84	0.00	-	0.84	0.84	0.84	0.84	0.00	0.42	0.00
Smokers	1772.33	0.21	-	0.84	0.00	-	0.84	0.84	-	0.84	0.00	0.00	0.00
People who practice physical exercise	1569.33	0.19	-	0.00	0.00	-	0.00	0.37	0.37	0.37	0.00	0.00	0.00
Obese children	304.00	0.11	-	-	0.00	0.07	0.00	0.07	-	-	0.00	0.00	0.00
Overweight children	304.00	0.11	-	-	0.00	0.07	0.00	-	-	-	0.00	0.00	0.00
Children with healthy eating	282.00	0.10	-	-	0.00	0.00	0.00	-	-	-	0.00	0.00	0.00
Children who practice physical exercise	253.67	0.09	-	-	0.00	0.00	0.00	0.06	-	-	0.00	0.00	0.00
	Average	0.07	0.00	0.56	0.00	0.02	0.10	0.15	0.40	0.42	0.00	0.02	0.00
	SD	0.08	0.00	0.48	0.00	0.03	0.27	0.29	0.42	0.41	0.00	0.10	0.00
	Median	0.02	0.00	0.84	0.00	0.00	0.00	0.02	0.37	0.37	0.00	0.00	0.00

CVI: Care Vulnerability Index; VCF: Vulnerability Correction Factor. Average of the population represented in the different measurements of the indicator in the study period and applied to adjust the score to the percentage of the population represented in the total study sample; SD: Standard Deviation.

**Table 6:** Maximum CVI and adjusted to population weight.

	Maximum CVI	CVI Adjusted to Population Weight
Cluster 1	0.90	0.18
Cluster 2	0.33	0.28
Cluster 3	1.05	0.18
Cluster 4	2.84	1.30
Cluster 5	0.02	0.02
	5.15	1.95

Maximum CVI: Vulnerability index results calculated with the maximum scores, without population adjustment; CVI Adjusted to population weight: Vulnerability index results calculated with scores adjusted by population through Vulnerability Correction Factor.

## Data Availability

Data are contained within the article.

## Appendix A

**Table A1:** Description of the sample of selected health indicators from the e-SOAP Specific Dashboard.

e-SOAP Code	Indicator	Definition	Formula	Measurement Frequency
5.04.95.	People with severe or total functional impairment (Barthel ≤ 60) with care plans.	Percentage of patients with severe or total functional impairment identified and followed in Primary Care who meet the Barthel index ≤ 60 and have a record of care plan follow-up.	(number of People with severe or total functional impairment (Barthel ≤ 60) with active care plans by nursing CIAS/number of People with severe or total functional impairment (Barthel ≤ 60) by nursing CIAS)*100	Quarterly
9.08.48.	Management of hypercholesterolemia in secondary prevention of ischemic heart disease	Percentage of patients in secondary prevention of ischemic heart disease with hypercholesterolemia controlled by nursing CIAS	number of patients > 35 years old with ischemic heart disease with an active history and with LDL recorded in the last 6 months whose last LDL level < 100/patients > 35 years old with CIAP of ischemic heart disease with an active history*100	Quarterly
9.70.76.	Chronic patients in health institutions with assigned NI (≥70 years)	Percentage of chronic patients ≥ 70 years who are identified as institutionalized with assignment of intervention level.	(number of patients with chronic pathology and assigned intervention level (≥70 years)/number of patients with chronic pathology (≥70 years))*100	Monthly
9.72.04.	Patients with ischemic heart disease and controlled blood pressure levels	Percentage of patients with ischemic heart disease identified and followed up in Primary Care who meet blood pressure (BP) control criteria.	(patients aged 15–79 years with ischemic heart disease with active clinical history with last blood pressure ≤ 140/90 in the last year/patients older than 15–79 years with diagnosis of ischemic heart disease with active clinical history in primary healthcare in the community of Madrid)*100	Quarterly
9.72.09.	Diabetic patients with adequate age-adjusted glycated haemoglobin control.	Percentage of diabetic patients aged 14 years or older identified and followed in Primary Care who meet Adequate glycated haemoglobin control criteria (last recorded HbA1c < 7% for ≤75 years and <8.0% for >75 years).	(patients >14 years with diabetes mellitus (DM) with active medical history whose last recorded HbA1 figure is <7% for ≤75 years and <8% for >75 years/patients > 14 years with a diagnosis of DM)*100	Quarterly
9.72.10.	Patients with diabetes and controlled blood pressure levels	Percentage of diabetic patients between 15 and 79 years of age identified and followed in Primary Care with followed in Primary Care with systolic blood pressure <140 mmHg and/or diastolic blood pressure < 90 mmHg.	(patients between 15–79 years old with active clinical history and whose last recorded figures are: Systolic blood pressure ≤ 140 and/or Diastolic blood pressure ≤ 90 mmHg/patients between 15–79 years old with active clinical history with diagnosis of DM)*100	Quarterly
9.72.19.	Patients with chronic kidney disease and controlled blood pressure levels adjusted for age and albuminuria.	Percentage of patients with chronic kidney disease (CKD) identified and followed in Primary Care who meet criteria for good blood pressure control (cardiovascular risk factor) as defined by the National Consensus Document.	(patients between 15 and 79 years old with CKD and active medical history who meet criteria for control of age-adjusted blood pressure and albuminuria/patients between 15 and 79 years old with CKD and active medical history)*100	Quarterly
9.72.27.	Patients with stroke and controlled blood pressure levels	Percentage of patients with stroke identified and followed up in Primary Care who meet blood pressure (BP) control criteria.	(Patients between 15 and 79 years with active medical history with diagnostic criteria of stroke with last blood pressure ≤ 140/90 in the last year/Patients between 15 and 79 years with active medical history with a diagnosis of stroke)*100	Quarterly
9.72.32.	Patients with heart failure and controlled blood pressure levels	Percentage of patients with heart failure (HF) identified and followed up in Primary Care who meet blood pressure (BP) control criteria.	(Patients between 45 and 79 years old with active medical history with diagnostic criteria of Heart Failure with last Blood Pressure ≤ 140/90 in the last year/Patients between 45 and 79 years old with diagnosis of Heart Failure with active medical history)*100	Quarterly
9.72.34.	Patients with arterial hypertension with age-adjusted blood pressure monitoring	Percentage of patients with hypertension identified and followed up in Primary Care who meet age-adjusted blood pressure (BP) control criteria.	(Patients older than 14 years with active medical history with diagnostic criteria of hypertension whose last BP ≤ 140/90 if age < 80 years or BP ≤ 150/90 if age ≥ 80 years in the last year/Patients older than 14 years with active medical history with a diagnosis of hypertension)*100	Quarterly
9.73.00.	People with healthy eating	Percentage of people over 14 years of age with a healthy diet (Mediterranean diet)	(persons over 14 years of age with active medical history with healthy eating (Mediterranean diet or healthy eating recorded)/total number of persons over 14 years of age with active medical history)*100	Quarterly
9.73.01.	Persons with risky alcohol consumption	Percentage of people over 14 years of age who engage in risky alcohol consumption	(persons over 14 years of age with an active medical history of risky alcohol consumption/persons over 14 years of age with an active medical history)*100	Quarterly
9.73.02.	Smokers	Percentage of smokers older than 14 years of age	(persons over 14 years of age with active medical history with smoking record in the last two years/persons over 14 years of age with active medical history)*100	Quarterly
9.73.05.	People who practice physical exercise	Percentage of people between 14 and 65 years of age who practice physical exercise	(persons between 15 and 64 years old with an active medical history who practice physical exercise (≥2.5 hours per week)/persons between 15 and 64 years old with an active medical history)*100	Quarterly
9.73.06.	Obese children	Percentage of children 2 to 14 years old with obesity	(children from 2 to 14 years old with active medical history with obesity medical record*100/children from 2 to 14 years old with active medical history)*100	Quarterly
9.73.07.	Overweight children	Percentage of children aged 2–14 years who are overweight.	(children aged 2 to 14 years with active medical history with overweight medical record*100/children aged 2 to 14 years with active medical history)*100	Quarterly
9.73.08.	Children with healthy eating	Percentage of children 4 to 14 years old with healthy diet	(children aged 4 to 14 years with active medical history with healthy eating/total children aged 4 to 14 years with active medical history)*100	Quarterly
9.73.09.	Children who practice physical exercise	Percentage of children aged 6 to 14 years who practice physical exercise.	(children from 6 to 14 years old with active medical history who practice physical exercise (≥3 h/week)/children from 6 to 14 years old with active medical history)*100	Quarterly
